# Increased grain weight conferred by *GW2* mutations in wheat does not translate into yield gains in multi-year field trials of near-isogenic lines

**DOI:** 10.1093/jxb/erag326

**Published:** 2026-07-02

**Authors:** James Simmonds, Pamela Crane, Sophie Eade, Aura Montemayor-Lara, Matt Kerton, Nicholas Bird, Phil Tailby, Peter Jackson, Duncan Warner, Charlotte Hayes, David Schafer, Cristobal Uauy

**Affiliations:** John Innes Centre, Norwich Research Park, Norwich NR4 7UH, UK; John Innes Centre, Norwich Research Park, Norwich NR4 7UH, UK; John Innes Centre, Norwich Research Park, Norwich NR4 7UH, UK; John Innes Centre, Norwich Research Park, Norwich NR4 7UH, UK; DSV UK Ltd, Top Dawkins Barn, Oxford, UK; KWS UK Ltd, Royston, UK; Limagrain UK Ltd, Woolpit, UK; NPZ UK Plant Breeding Ltd (Formerly LS Plant Breeding Ltd), Cambridge, UK; Syngenta UK Ltd, Jealott's Hill International Research Centre, Bracknell, UK; Elsoms Seeds Ltd, Spalding, UK; RAGT Seeds Ltd, Walden, UK; John Innes Centre, Norwich Research Park, Norwich NR4 7UH, UK; The University of Adelaide, Australia

**Keywords:** Grain weight, mutants, *TaGW2*, trade-off, *Triticum aestivum*, wheat, yield

## Abstract

Multiple studies have identified genes affecting grain morphology, yet their capacity to deliver yield gains under field conditions remains unclear. We performed a multi-year, multi-location factorial evaluation of *GRAIN WIDTH2* (*TaGW2*) mutants in hexaploid wheat using BC_4_ near-isogenic lines, sowing density treatments, and semi-dwarfing *RHT1* backgrounds. Loss-of-function mutations in *TaGW2* increased grain size and thousand grain weight (TGW) additively, with the *aaBBDD* single mutant showing the most stable single-locus effect, while the *aabbdd* triple mutant achieved ∼20% higher TGW across 12 field trials. However, overall grain yield remained unchanged or slightly reduced, reflecting a compensatory trade-off with grain number. Spike phenotyping of both main and secondary tillers showed comparable increases in TGW and spike yield despite fewer grains per spike, indicating that limited yield gain primarily reflects reduced spike number per unit area rather than decreased spike-level productivity. Effects were stable across sowing densities, whereas interactions with semi-dwarfing alleles were allele specific: *RHT-B1b* partially suppressed TGW gains and accentuated yield penalties, whereas *RHT-D1b* maintained the large-grain phenotype and productivity. Across experiments, the *TaGW2-A1D1* double mutant increased TGW (∼14%) while maintaining yield stability, identifying it as a promising genotype for breeding. We conclude that *TaGW2* is a reliable modifier of grain size but not yield in isolation.

## Teaser

Multi-year field trials reveal that *TaGW2* mutations increase grain size but not yield, guiding breeding strategies to optimize the grain size–grain number trade-off.

## Introduction

Wheat (*Triticum aestivum* L.) is a staple crop providing a major source of calories and protein for the global population. As global wheat demand continues to outstrip the rate of yield improvement (Fischer *et al*., 2014; FAO, 2018), future food security faces considerable challenges, especially under ever-increasing environmental pressures. Yield improvements are also at the heart of initiatives to reduce greenhouse gas emissions in agriculture; for example, the UK Seventh Carbon Budget calls for a 16% increase in crop yields as a key driver to halve CO_2_ emissions by 2050 (Climate Change Committee, 2025). Accordingly, the identification, functional characterization, and incorporation of genes and alleles that enhance yield represent key priorities for breeding programmes worldwide.

Grain yield in wheat is a complex polygenic trait, determined primarily by its three major components: the number of spikes per area, the number of grains per spike, and the average weight of individual grains. Wheat yield improvements have traditionally relied on increasing grain number per unit area, achieved through the combined effects of genetic selection and improved agronomic practices (Calderini *et al*., 1995; Slafer *et al*., 2021). Numerous studies have proposed grain size and weight as alternative avenues for yield improvement (Simmonds *et al*., 2014; Brinton *et al*., 2017; Adamski *et al*., 2021; Calderini *et al*., 2021; Wei and Jiao, 2024). Although historical increases in grain size have contributed little to yield gains during breeding (Philipp *et al*., 2018), there is a clear rationale for focusing efforts on gene discovery for grain morphometric traits. Grain size is the final yield component to be determined, is stably inherited, and exhibits higher heritability than overall grain yield, making it an attractive target for genetic improvement.

One gene that has attracted significant attention is *GRAIN WIDTH 2* (*GW2*), encoding a RING-type E3 ubiquitin ligase that negatively regulates grain size in rice (Song *et al*., 2007) and whose function seems conserved across cereals (Li *et al*., 2010; Tao *et al*., 2017; Long *et al*., 2024). Wheat orthologues of *GW2* (*TaGW2*) have been shown to play similar roles, with targeted loss-of-function mutations resulting in larger grains and increased thousand grain weight (TGW) by promoting cell expansion in the grain pericarp (Simmonds *et al*., 2016; Zhang *et al*., 2018). Previously we demonstrated in BC_1_F_3_ lines grown in controlled-environment rooms that mutations from the three wheat homoeologues act in an additive manner, with grain size effects ranging from ∼5.5% in the single mutants, to 13.4% in the double mutants, and to 20.7% in the full knockout (triple) (Wang *et al*., 2018). Subsequently, additional studies confirmed these effects in CRISPR-induced mutants (Zhang *et al*., 2018; Achary and Reddy, 2021; Kis *et al*., 2024).

However, the effect of *TaGW2* (referred to hereafter as *GW2*) mutations on final yield remains context dependent and may vary across genetic backgrounds and environments due to compensatory effects on grain number and source–sink dynamics (Vicentin and Calderini, 2025, Preprint). The extent to which these improvements translate into consistent yield advances is rarely reported, especially from field-based yield experiments, across multiple sites and years.

This study aims to evaluate the relationship between grain weight and yield in *GW2* near-isogenic lines (NILs) across multiple field environments and years. We utilized BC_4_ NILs developed from wheat *GW2* TILLING (Targeting Induced Local Lesions in Genomes) mutations in a UK elite genetic background (Simmonds *et al*., 2016; Wang *et al*., 2018) to assess the phenotypic effects of the gene while minimizing confounding variation from unrelated genomic regions. We compared the effects of individual *GW2* homoeologues as well as their combined contributions, providing insights into the additive and potentially compensatory roles of these genes in wheat yield determination. In addition, we evaluated the effects of *GW2* homoeologues under contrasting *REDUCED HEIGHT 1* (*RHT1*) alleles, given that semi-dwarfing *RHT1* alleles are known to negatively affect grain size but have positive effects on yield and grain number (Keyes and Sorrells, 1989; Flintham *et al*., 1997; Jobson *et al*., 2018). Semi-dwarfing alleles *Rht-B1b* and *Rht-D1b* are widely deployed in UK and global breeding programmes for lodging control and yield stability. As these alleles underpin the genetic background of most commercial varieties, any practical application of *GW2* alleles will depend on their ability to function effectively in a semi-dwarf context. Through understanding the pleiotropic effects of *GW2* alleles on yield components, we can assess the stability of *GW2*-associated phenotypes and their implications for wheat yield improvement strategies.

## Materials and methods

### Plant materials

We previously identified mutations in the three *GW2* homoeologues from the tetraploid *Triticum turgidum* cv Kronos and hexaploid *T. aestivum* cv Cadenza TILLING populations (Simmonds *et al*., 2016; Krasileva *et al*., 2017; Wang *et al*., 2018) (Supplementary Table S1). Mutations in the A and B genomes were identified in the Kronos populations, and the D genome mutation came from Cadenza. Mutations were individually crossed to cv Paragon and the F_1_ were then intercrossed to combine all mutations into a single plant. The presence of mutations was confirmed through marker-assisted selection with single nucleotide polymorphism (SNP)-specific KASP (Kompetitive Allele-Specific PCR) markers (Supplementary Table S2). Heterozygous triple mutant plants (*AaBbDd*) were self-pollinated to produce homozygous BC_1_F_2_ plants as described in Wang *et al*. (2018). The homozygous BC_1_ plants were backcrossed again, and BC_2_F_1_ plants were self-pollinated and homozygous wild-type (WT) and mutant plants for all homoeologue combinations were extracted at BC_2_F_2_. The BC_2_ NILs were utilized in field Experiment 1 (2018). Backcrossing continued and advanced to BC_4_F_2_ NILs to minimize off-target regions, and these were sown in Experiments 2–5.

As Paragon does not carry either of the main *RHT1* semi-dwarfing alleles utilized in the UK (*RHT-D1b* or *RHT-B1b*), we wanted to assess the interaction of the full *GW2* knockout with these alleles. BC_4_  *GW2* triple mutant NILs were crossed to Paragon *RHT-D1b* and Paragon *RHT-B1b* NILs, developed as part of the ‘Paragon Library’ through the Wheat Genetic Improvement Network (WGIN). The F_1_ seed were self-pollinated and, after screening with KASP markers, homozygous F_2_ lines with the *GW2* triple mutant alleles or three WT alleles, and with either *RHT-D1b*, *RHT-B1b*, or WT alleles at *RHT1*, were selected. For all genotypes, we developed two independent crossing streams. Analyses, however, were performed by genotype and therefore streams were combined.

Seed of the Paragon BC_4_  *GW2* single homoeologous mutants and the Paragon BC_4_  *GW2* triple mutant used in this study are available via the JIC Germplasm Resources Unit (https://seedstor.ac.uk/) with the following accession numbers WM0011 (A genome), WM0005 (B genome), WM0007 (D genome), and WM0015 (triple mutant). Seed of the Paragon *RHT* NILs and Paragon *GW2×RHT* NILs are also available to order; WParNil0077 (Paragon *RHT-D1b*), WParNil0078 (Paragon *RHT-B1b)*, WM0325 (Paragon *GW2_triple+RHT-D1b*), and WM0326 (Paragon *GW2_triple+RHT-B1b*).

### Field experiments

All field trials, apart from Experiment 4, were sown at The Morley Agricultural Foundation, Wymondham, Norfolk, UK (52.55421, 1.03274). BC_2_/BC_4_ NILs were sown in the autumn in 2018 and 2020 (Experiments 1 and 3), respectively, but in early spring in 2020 (Experiment 2) due to unfavourable weather/soil conditions in autumn 2019. Experiments 1, 2, 3, 5, and 6 were sown in yield plots 6 m×1.2 m, aiming for a target population of 275 plants m^−2^, consisting of eight rows with 0.13 m spacing. Trials were sown in a randomized complete block design with either three blocks (Experiment 1) or five blocks (Experiments 2, 3, 5, and 6) and with two or three NILs for each genotype developed from independent crossing streams. Plot ends were removed pre-harvest to leave a 4 m×1.2 m combinable plot area.

In 2020, the BC_4_ NILs of the triple mutant (*aabbdd*) and WT (*AABBDD*) were also sown in multi-site trials as part of the ‘Designing Future Wheat’ (DFW) Breeders Toolkit (Experiment 4). These were sown in three replicate trials at multiple locations across England with the breeding companies DSV, Elsoms, KWS, Limagrain, NPZ UK, and Syngenta.

To test the performance of the *GW2* BC_4_ NILs at different planting densities, we sowed a trial in 2022 (Experiment 5) at three seed rates, the standard 275 plants m^−2^, an extremely high seed rate of 500 plants m^−2^, and a low seed rate aiming for 125 plants m^−2^.

To test if *GW2* interacted with *RHT1*, we developed *GW2×RHT-B1b/D1b* NILs and these were also sown in 2022 at the standard seed density (275 plants m^−2^). All trials listed above received standard farm pesticide and fertilizer applications to reproduce commercial practice.

The locations and details of all field experiments are highlighted in Supplementary Table S3.

### Phenotypic assessments

The following phenotypic assessments were made, with corresponding Crop Ontology classifications and a full breakdown of traits scored in each experiment provided in Supplementary Table S4. Heading date was assessed as the number of days from 1 May until three-quarters of the canopy had reached thee-quarter emergence (Zadoks growth stage 57) (Zadoks *et al*., 1974). Maturity was scored when the green colour in the peduncles had been lost in 50% of the plot, and was calculated as the number of days from 1 May until maturity. The grain-filling period was calculated as the difference in days between heading and maturity. Tiller number was assessed by counting the number of fertile tillers along two 1 m rows per plot and calculating the average, always using the second row from each side of the plot. Crop height was measured from the soil level to the average height of the canopy.

For Experiments 1–3, prior to harvest, 10 main spikes were sampled from each plot for spike yield components including spikelet number, rudimentary basal spikelets, seeds/spike, yield/spike, and seeds/spikelet. For Experiments 5 and 6, two 1 m strips were marked out prior to tiller counts to ensure that the same area was counted at both growth stages. These sections were then hand-pulled from each plot to enable the analysis of the above spike yield components to be carried out separately for main tiller and secondary tillers. After separation, all main spikes and secondary spikes were counted, and the ratio of secondary spikes to main spikes was calculated. Of these, we randomly sampled five main spikes and five secondary spikes from each 1 m strip to analyse for spike yield components (10 total spikes per plot).

All plots were combined and the following measurements recorded: plot yield, grain moisture, and hectolitre weight. Post-harvest grain samples were taken from each plot, and morphometric measurements (grain width, length, area, and TGW) were recorded from ∼500 grains per sample using the MARViN grain analyser (GTA Sensorik GmbH, Germany). In addition, seed was analysed for protein, starch, fibre, dry gluten content, and grain hardness using the Perten DA 7250 NIR Analyser (PerkinElmer, Perten Instruments, Hägersten, Sweden).

All raw data from Experiments 1–6 have been deposited on Zenodo (Simmonds *et al*., 2026).

### Statistical analysis

Experiments 1–3 (2019–2021) were analysed separately using fixed-effects ANOVA models of the form Trait∼Genotype+Block, with both factors treated as fixed. Tukey’s Honest Significant Difference (HSD) test (α=0.05) was used for post-hoc comparisons among genotypes. To assess overall effects across years, a combined two-way ANOVA (Trait∼Genotype+Year+Genotype×Year) was performed to evaluate main effects and interactions.

For Experiment 4, data from multiple trial locations were analysed both collectively (Trait∼Genotype+Location+Genotype×Location) and separately within each site using one-way ANOVA models (Trait∼Genotype+Block).

In Experiment 5, effects of genotype, seed rate, and their interaction were tested using a multi-factor ANOVA (Trait∼Genotype+SeedRate+Block+Stream+Genotype×SeedRate). Tukey’s HSD test identified significant differences among genotypes and seed rates. An additional one-way ANOVA compared all genotype×seed rate combinations (factor: GW2_SR).

For Experiment 6, a two-way ANOVA (Trait∼Rht_Allele+GW2_ABD+Block+Rht_Allele×GW2_ABD) assessed main and interaction effects, followed by Tukey’s HSD test. A separate one-way ANOVA examined the combined effects of *RHT_GW2* with replication included.

All analyses were conducted in R using base functions (aov) (RStudio Team, 2025) and the HSD.test() function from the agricolae package (de Mendiburu, 2019).

## Results

### Individual GW2 homoeologues confer modest increases in grain size and weight

All combinations of the BC_4_ single, double, and triple mutant NILs were evaluated in three field trials over consecutive years (2019–2021; Experiments 1–3) to assess their effects on grain size, grain weight, final grain yield, and yield components.

Among the single mutants, *GW2_A1* (*aaBBDD*) was the highest performing, increasing TGW by an average of 6.7% relative to the WT control NIL (range 4.4–7.4%; Fig. 1; Supplementary Table S5), consistent with previously reported results (Simmonds *et al*., 2016). Although only significant in 2020, combined analysis across all three trials indicated that *GW2_A1* was the only single mutant with a significant benefit on TGW. This was due to significant increases in both grain width and length. *GW2_B1* (*AAbbDD*) also exhibited significantly increased grain length in 2019 and in the overall analysis, but its 1.1% increase in yield compared with the WT was non-significant. *GW2_D1* (*AABBdd*) showed no significant differences in TGW or grain size parameter compared with the WT, supporting previous findings (Wang *et al*., 2018).

**Fig. 1.**
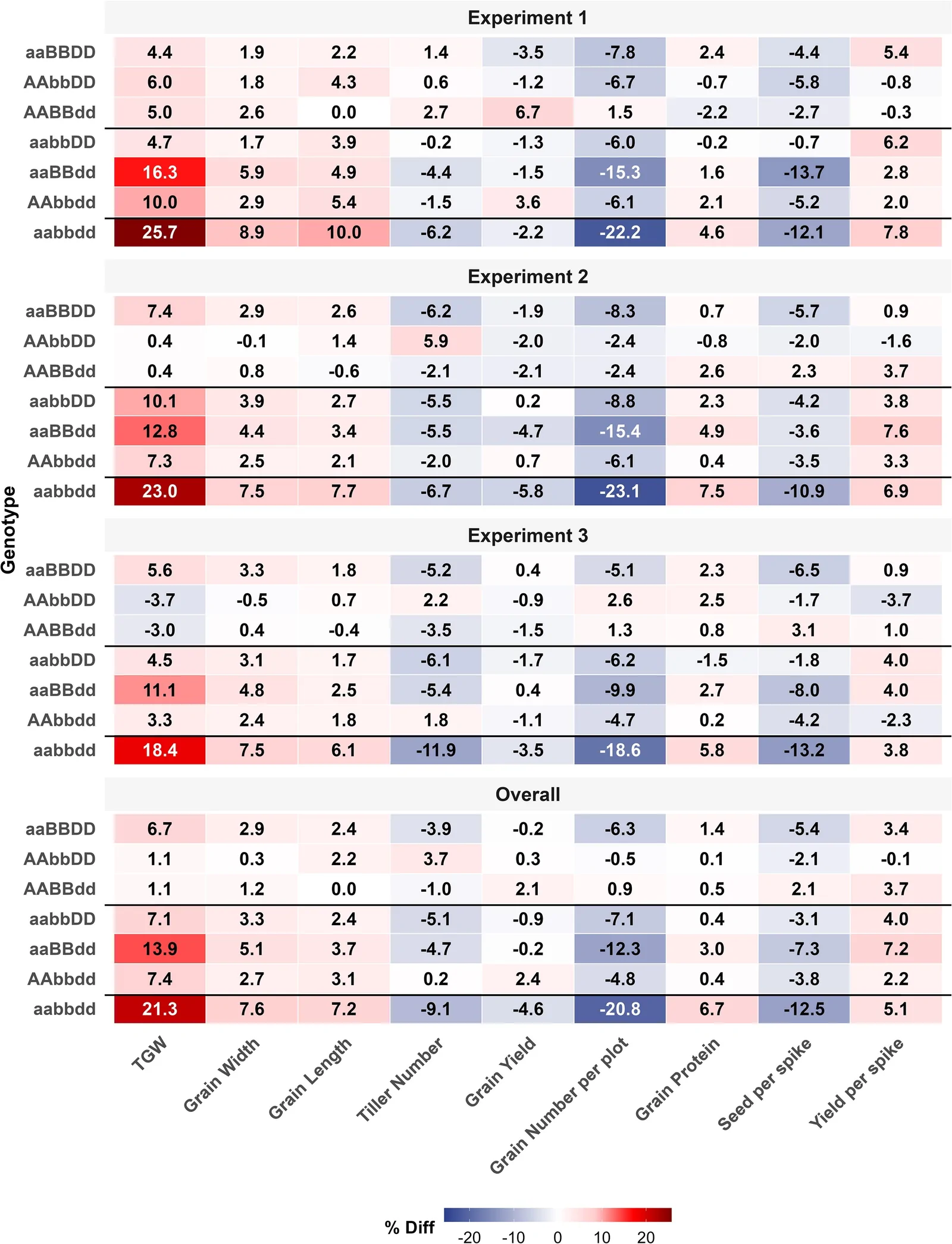
Heatmap showing the percentage differences of *GW2* mutant NILs relative to the wild-type control in Experiments 1–3 (2019–2021). Single (*aaBBDD*, *AAbbDD*, *AABBdd*), double (*aabbDD*, *aaBBdd*, *AAbbdd*), and triple (*aabbdd*) *GW2* mutant NILs are compared with the wild-type control NIL across individual experiments and the overall analysis. Cell shading represents the direction and magnitude of the percentage differences relative to the control. Positive values indicate increases and negative values indicate decreases. Statistical significance is not displayed on the heatmap but is provided in Supplementary Table S4.

For other traits, the only significant differences from the WT were observed for *GW2_A1*, which showed reduce calculated grain number per plot (cGNPlot) in 2020 and reduced cGNPlot and seeds per spike in the overall analysis. None of the single mutants showed a significant effect on overall grain yield (Fig. 1; Supplementary Table S5).

### *GW2* homoeologues act additively to increase grain size and weight (Experiments 1–3)

Combinations of *GW2* mutants were assessed for their combined effect on productivity traits across the three field trials (Experiment 1–3). For TGW, all double mutants (*aabbDD*, *aaBBdd*, and *AAbbdd*) showed significant increases compared with the WT. Among them, *GW2_A1D1* (*aaBBdd*) had the largest and most consistent effect, with TGW increases in all three experiments and an overall increase of 13.9% compared with ∼7% for the other double mutant combinations. The triple mutant *GW2_A1B1D1* (*aabbdd*) showed a further increase, with TGW 21.3% higher than in the WT (*P*<0.001) and significantly higher than in any double mutant (Fig. 1; Supplementary Table S5). This increase was driven by proportional increases in both grain width and length, consistent with previous findings (Wang *et al*., 2018).

Despite these marked increases in grain size and weight, grain yield in the double mutants did not differ significantly from the WT. Analysis of yield components revealed compensatory reductions on a series of traits that contribute to yield, including final tiller number (−0.2% to −5.1%), grains per plot (−4.8% to −12.3%), and seeds per spike (−3.1% to −7.3%). These negative effects were further exacerbated in the triple mutant, resulting in a significant 4.6% reduction in grain yield relative to the WT NIL (Fig. 1; Supplementary Table S5).

Across all measured traits, the triple mutant showed significant increases in TGW (21.3%), grain width (7.6%), grain length (7.2%), grain protein concentration (6.7%), and dry gluten content (7.9%). However, several yield component traits were significantly decreased, including tiller number (−9.1%), hectolitre weight (−2.0%), grains per plot (−20.8%), spikelet number (−3.4%), seeds per spike (−12.5%), and seeds per spikelet (−8.6%) (Fig. 1; Supplementary Table S5).

Interestingly, despite the triple mutant producing spikes with fewer seeds, the total grain weight per spike (yield per spike) was consistently higher than in the WT, ranging from 3.8% to 7.8%, although the differences were not significant. These findings highlight a trade-off between grain size and yield components, which motivated further experiments (Experiments 5 and 6) to dissect the basis of this relationship.

### Multi-site breeders trials confirm consistent effects of GW2 mutants (Experiment 4)

To validate the results from the multi-year, single-site trials, we conducted a multi-site trial at the field stations of six UK wheat breeders across England (Experiment 4) comparing the *GW2_A1B1D1* triple mutant (*aabbdd*) and the WT (*AABBDD*). Data were analysed both for individual sites and across all locations (Supplementary Table S6). TGW was significantly increased at all sites, with an average gain of 18.2% relative to the WT, confirming the robust and stable effect of *GW2* on grain weight observed in Experiments 1–3. Grain protein concentration (4.6%) and dry gluten content (8.8%) were also significantly higher in the triple mutant (supporting the data from Experiments 1–3) and showed consistent improvement across locations, although not all sites were statistically significant. Across all sites, grain yield was unchanged between genotypes (8.912 t ha^−1^ for the triple mutant versus 8.905 t ha^−1^ for the WT; *P*=0.971). Yield was significantly decreased at one site (Elsoms −9.6%; *P*=0.02), whereas in all other locations the triple mutant showed slight, non-significant yield increases (ranging from 0.5% to 7.8%). Based on the yield and TGW values, the calculated grain number was significantly reduced by 15.4% across environments. Thus, the multi-site breeder trials confirmed that *GW2*-mediated increases in grain size and weight are stable across diverse UK environments, but yield remains unchanged due to a consistent trade-off with grain number.

### Grain size and yield are unaffected by sowing density (Experiment 5)

To further investigate the nature of this trade-off, we grew the *GW2-A1B1D1* triple mutant and WT NILs at three sowing densities: low (125 plants m^−2^), standard rate (275 plants m^−2^), and high (500 plants m^−2^) in Experiment 5. This experiment was designed to manipulate tillering potential, promoting high tillering in the low seed rate and reduced tillering in the high seed rate, and to evaluate whether *GW2* effects on productivity were influenced by plant density. Previously we observed that tillers m^−1^ assessed pre-harvest (GS87) were consistently lower in the triple mutant in all three experiments, and 9.1% lower on average (Fig. 1; Supplementary Table S5). For this experiment, we additionally measured plant establishment and tiller number at GS32 (just prior to tiller abortion), and calculated the tillers per plant at each stage and the rate of tiller abortion.

Across genotypes, sowing density had the expected, significant effect on plant establishment: the low, standard, and high seed rate produced 22.2, 32.4, and 45.6 plants m^−1^, respectively (Supplementary Table S7). Tiller production was also successfully manipulated, with tiller numbers per plant of 3.3 (low), 1.9 (standard), and 1.4 (high).

Interestingly, we discovered that the triple mutant exhibited reduced plant establishment at all three seed rates, significantly so at the high density (Fig. 2A; Supplementary Table S7). At GS32, tiller counts were significantly lower in the triple mutant at the high seed rate (119.2 tillers m^−1^ versus 133.0 tillers m^−1^ for the WT), but not at the standard sowing rate. At pre-harvest, the expected reduction in tiller numbers was evident at both the standard (−13%) and high (−10%) seed rates, but not at the low seed rate. Despite evidence of some subtle interactions (Fig. 2A), tiller abortion rates were unaffected by *GW2* (∼43% in both genotypes), and increased significantly with higher sowing densities.

**Fig. 2.**
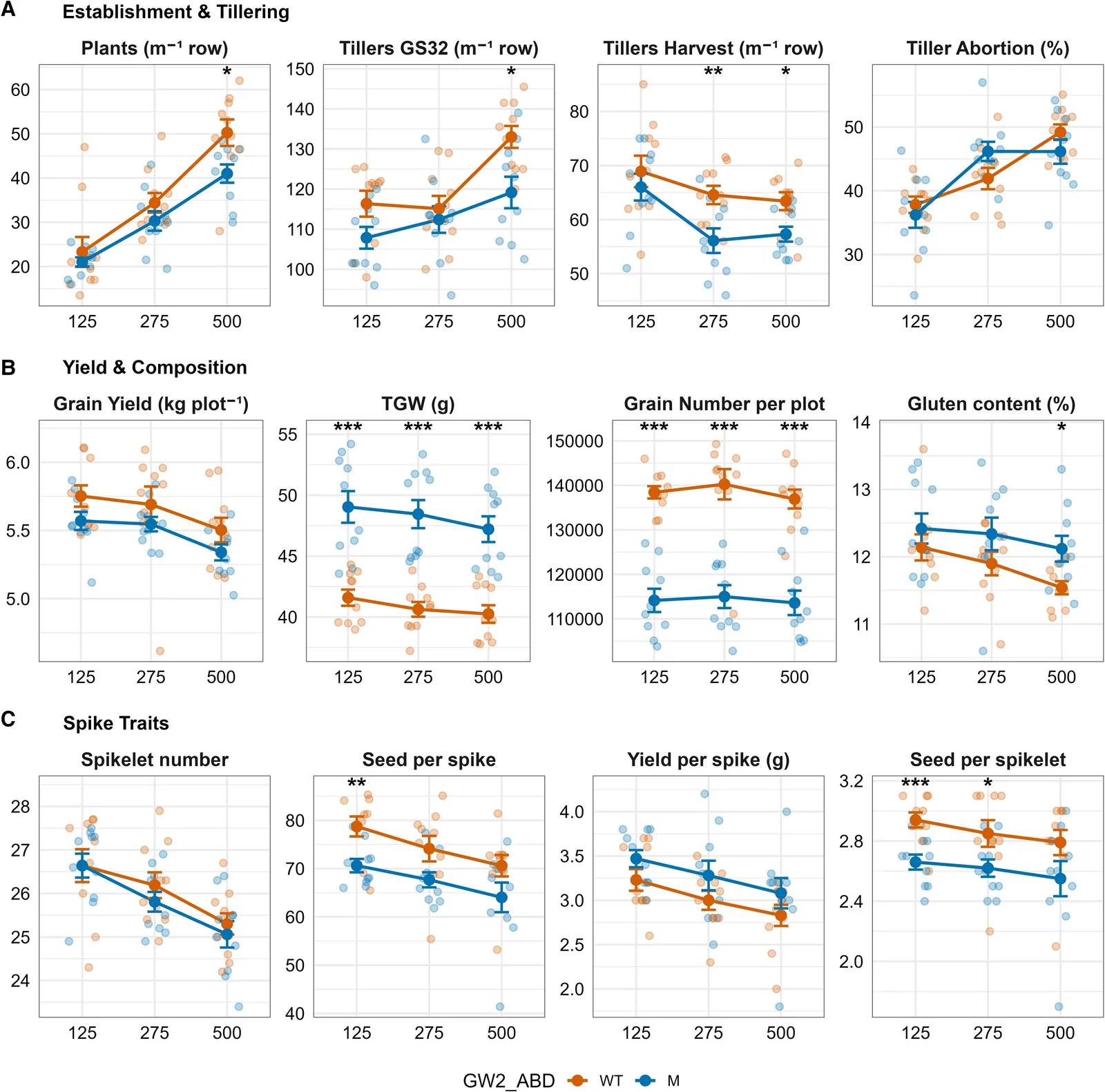
Interaction plots of selected traits for BC_4_  *GW2* NILs sown at three seed densities (125, 275, and 500 plants m^−2^) in Experiment 5. Lines and points represent the two genotypes (WT and M), as indicated in the legend. Mean values (filled circles) are presented with SE bars. Significance levels between WT and triple mutant (M) NILs at each seed rate were determined by two-sample *t*-tests: ***P<0.001, **P<0.01, *P<0.05; no symbol indicates *P*≥0.05. Panels represent groups of traits: (A) establishment and tillering (recorded from two 1 m rows per plot), (B) yield and grain composition, and (C) spike traits (measured from 10 sampled main spikes).

Seed rate had little impact on the effect of *GW2* on yield and grain quality traits (Fig. 2B). TGW was significantly increased in the triple mutant at all three seed rates (mean 18.2%), while grain yields were significantly lower when analysed across all seed rates (−2.9% relative to the WT). Interestingly, within genotypes, yields were similar at the low and normal seed rates, but significantly lower at the high density. Spike yield components were also unaffected by seed rate (Fig. 2C); consistent with previous experiments, the triple mutant produced fewer seeds per spike and seeds per spikelet, but overall increased yield per spike from main spikes. Taken together, these results indicate that *GW2* effects on grain size and yield are stable across sowing densities, with no evidence of genetic interaction between *GW2* allelic status and plant density.

### No compensatory effect from secondary tillers (Experiment 5)

Analysis of spike yield components indicated that in main spikes, mutations in *GW2* led to an inverse relationship between grain size and grain number, yet yield per spike was consistently higher. However, this advantage was not reflected in total plot yield. We therefore asked whether our focus on main spikes might have biased the results, and whether secondary tillers might compensate for reduced tiller number or altered spike traits. Given that tillering was experimentally manipulated by sowing density, we expected a higher proportion of secondary spikes to main spikes at low density and fewer at higher density.

To test this, we phenotyped 10 main spikes and 10 secondary spikes per plot for both genotypes across the three sowing densities. Analysis of the percentage difference between the triple mutant and WT revealed no significant differences in the response of main versus secondary spikes for any major spike yield component (Fig. 3A; Supplementary Table S7). The relative percentage differences between *GW2* alleles were highly consistent across spike types and seed rates: both main and secondary spikes exhibited higher TGW, fewer seeds per spike and per spikelet, and overall higher spike yield compared with the WT.

**Fig. 3.**
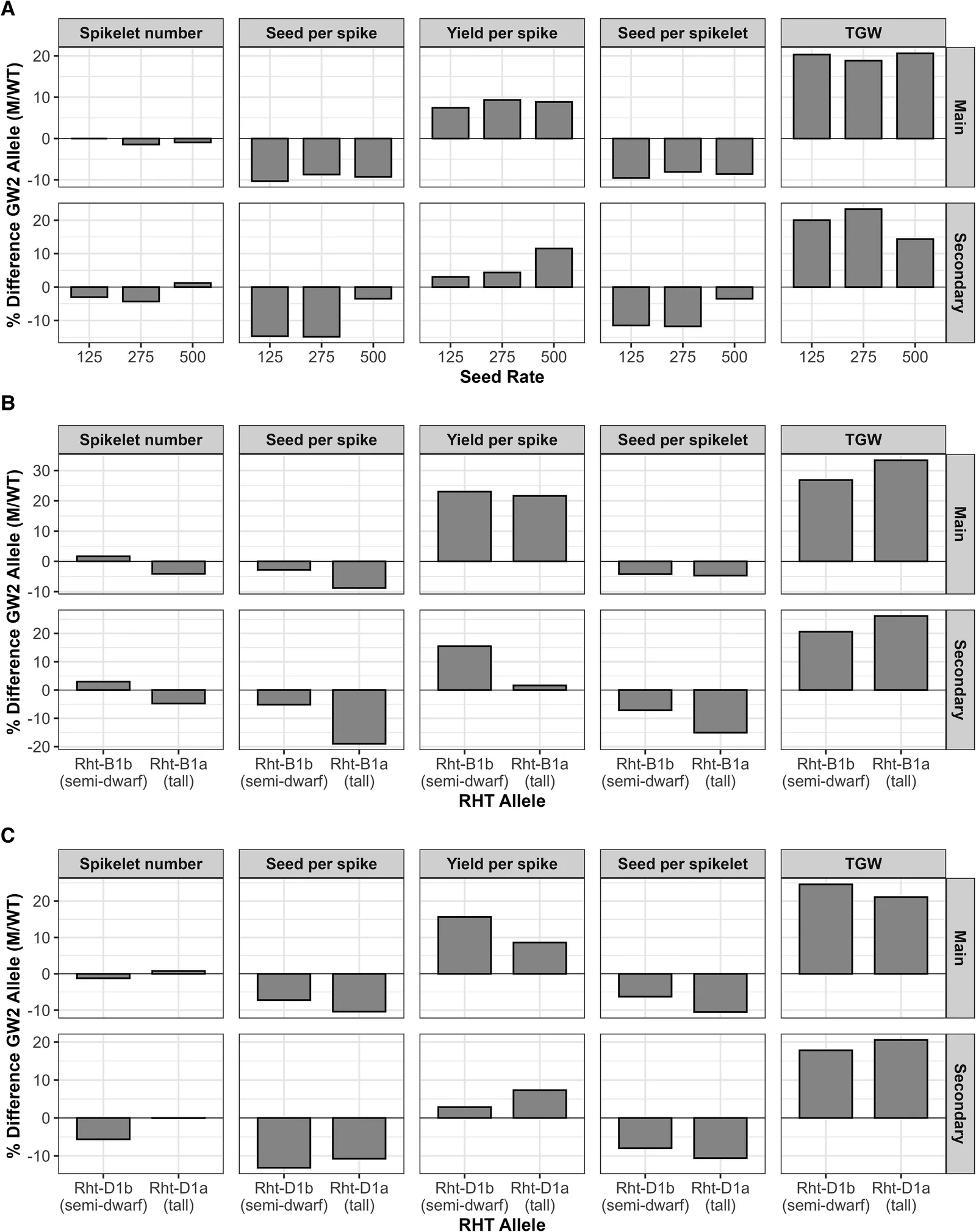
Divergent bar charts showing the percentage difference between *GW2* mutant and WT NILs for selected traits. (A) BC_4_  *GW2* NILs from Experiment 5; (B) BC_3_  *GW2×RHT-B1* NILs from Experiment 6, and (C) BC_3_  *GW2×RHT-D1* NILs from Experiment 6. Bars represent the percentage difference between triple mutant and WT NILs for both main and secondary spikes across seed rates (A) or the *RHT* allele background (B, C). Two-way ANOVA was performed for each trait to test for interactions between spike type (main versus secondary) and genotype. No significant differences were detected (*P*≥0.05) for any trait.

As intended, the ratio of secondary to main spikes (ST_MT_EarN_r) varied significantly with seed rate; 2.3, 1.0, and 0.5 secondary spikes per main spike at the low, standard, and high densities, respectively, but showed no interaction with *GW2* (*P*=0.758). This indicates that the main and secondary spikes responded similarly. Although the magnitude of effects was slightly smaller in secondary spikes, the overall yield per spike still increased, suggesting that the reduction in total grain yield in *GW2* mutants primarily results from fewer tillers or spikes, rather than from altered spike-level productivity.

### GW2 phenotype is unaffected by *RHT1* (Experiment 6)

Given that the parental background (cv. Paragon) carries the WT alleles at both major dwarfing loci (*RHT-B1a* and *RHT-D1a*), we developed BC_3_  *GW2×RHT-B1/D1* NILs and evaluated them in Experiment 6. Previous studies have demonstrated that *RHT* alleles typically reduce grain size but increase grain number, thereby enhancing overall yield (Keyes and Sorrells, 1989; Flintham *et al*., 1997; Jobson *et al*., 2018). Conversely, *GW2* mutations decrease grain number and increase seed size. This experiment therefore provided an opportunity to investigate the potential interaction between these contrasting genetic effects.

As expected, crop height was significantly reduced in lines carrying *RHT-B1b* (−11%) and *RHT-D1b* (−15%), confirming the presence of the semi-dwarfing alleles (Supplementary Table S8A, B).

For TGW, we observed the expected reductions in both *RHT-B1b* (Fig. 4A; Supplementary Table S8A) and *RHT-D1b* (Fig. 4B; Supplementary Table S8B) backgrounds compared with the WT, consistent with the known effects of *RHT* on grain size, with *GW2* performing significantly worse in the presence of *RHT-B1*. When analysed independently of *GW2* status, TGW decreased significantly by 11% and 6% for *RHT-B1b* and *RHT-D1b*, respectively. However, a significant interaction was observed between *RHT-B1* and *GW2* (*P*<0.02): the *GW2* triple mutant*×RHT-B1a* displayed a significant TGW increase when in a *RHT-B1b* semi-dwarf background. No such interaction was detected with *RHT-D1b*, where TGW differences between *GW2* mutant and WT lines were proportionally similar regardless of *RHT-D1* status.

**Fig. 4.**
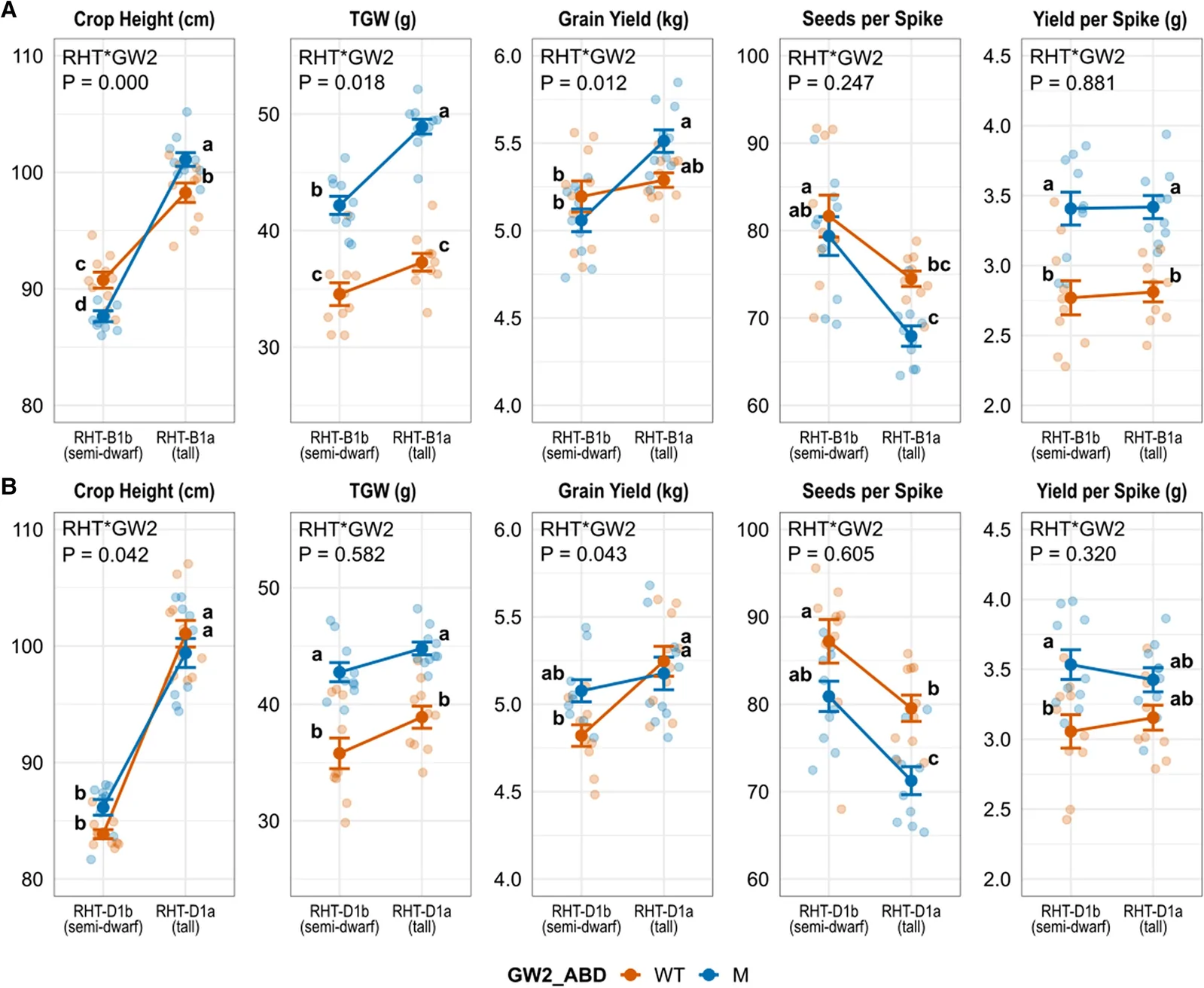
Interaction effects of *GW2* and *RHT* alleles on selected traits from Experiment 6. (A) BC_3_  *GW2×RHT-B1* NILs and (B) BC_3_  *GW2×RHT-D1* NILs. Lines and points represent the two genotypes (WT and M), as indicated in the legend. Mean values (filled circles) are presented with SE bars. Letters adjacent to each mean indicate groupings from post-hoc Tukey comparisons (α=0.05) based on an ANOVA of combined genotype factor (RHT Allele_GW2). Interaction *P*-values from a two-way ANOVA (RHT Allele×GW2) are displayed above each facet.

Significant interactions were also detected for grain yield, although these differed between *RHT-B1* and *RHT-D1* (Fig 4A, B). The *GW2* mutant yielded significantly less in the presence of *RHT-B1b*, whereas productivity was maintained with *RHT-D1b*. In both cases, the introduction of a semi-dwarfing allele reduced overall yield relative to the WT (Supplementary Table S8A, B).

No significant interactions were detected between *GW2* and *RHT1* for the two main spike yield components, seed number per spike (SSpk) and yield per spike (YldSpk). The expected trends were observed: semi-dwarfing *RHT1* alleles increased seed number per spike, while *GW2* mutants reduced it. Yield per spike was unaffected by *RHT1* but was elevated in the *GW2* mutant background.

Together, these results suggest that *GW2*-mediated increases in grain size are partially suppressed in the presence of semi-dwarfing *RHT1* alleles, particularly *RHT-B1b*. However, *RHT-D1b* did not negatively impact productivity in the *GW2* mutant background. This finding is of practical importance, as most UK cultivars utilize *RHT-D1b* to reduce plant stature.

### Secondary spike analysis confirms that spike yield is higher in all spikes (Experiment 6)

Phenotyping of the main and secondary spikes, as described in Experiment 5, was repeated for the *RHT-B1b* (Fig. 3B; Supplementary Table S8A) and *RHT-D1b* (Fig. 3C; Supplementary Table S8B) NILs. Consistent with previous results, no significant differences were detected between the main and secondary spikes when comparing *GW2* alleles. Thus, all spikes from *GW2* triple mutants contributed similarly to overall grain yield. Consequently, any reductions in total yield observed in these lines are likely to be attributable to decreased spike number per unit area rather than differences in per-spike productivity.

## Discussion

This study provides a comprehensive field-based evaluation of *GW2* in hexaploid wheat, combining multi-location, multi-year, and factorial experiments to elucidate its contributions to grain morphology, yield components, and agronomic performance. Consistent with previous reports (Simmonds *et al*., 2016; Wang *et al*., 2018; Zhang *et al*., 2018), mutations in *GW2* homoeologues increased grain size and TGW in an additive manner, confirming a conserved negative regulatory role for *GW2* in grain development in cereals (Song *et al*., 2007; Li *et al*., 2010; Kis *et al*., 2024). The single-copy *A* genome mutant (*aaBBDD*) produced the most stable TGW gains among single mutants (Fig. 1; Supplementary Table S5), while the combined triple mutant (*aabbdd*) achieved an average 20% increase in TGW across 12 field experiments (Supplementary Tables S5–S8). The stability of this effect across six breeder trials across a range of UK locations and sites underpins the robustness of *GW2* enhancement of grain weight (Supplementary Table S6).

However, despite substantial increases in TGW, overall grain yield at the plot level was not significantly altered in the *GW2* triple mutant and was occasionally significantly decreased (Fig. 1; Supplementary Tables S5–S8). This reflects the well-documented compensatory trade-off between grain size and grain number (Calderini *et al*., 1995; Fischer *et al*., 2014; Xie and Sparkes, 2021), where reduced grain number per spike and decreased spike numbers per unit area collectively offset the gains in individual grain weight. Comparable compensatory responses were also reported in a parallel set of field studies performed in Chile, where the *GW2* mutants displayed increased grain weight but reduced grain number across environments and sowing densities (Vicentin and Calderini, 2025, Preprint).

Physiologically, this trade-off probably arises from overlapping developmental windows for grain number determination and ovary growth. *GW2* loss of function accelerates and enhances early carpel growth, increasing ovary weight during booting and anthesis (Simmonds *et al*., 2016; Vicentin *et al*., 2024). In doing so, it appears to limit the total number of developing grains, probably through altered assimilate partitioning or reduced floret fertility. Our data show that, despite reductions in seed number per spike, yield per spike was consistently increased in the *GW2* triple mutant. This indicates that productivity at the spike level is enhanced; however, at the plant or canopy scale, overall grain yield remains constrained, presumably due to a reduced number of spikes per unit area. These findings are consistent with the ‘sink limitation’ hypothesis for wheat yield determination (Slafer *et al*., 2021, 2023), which proposes that yield stability is maintained through developmental compensation among yield components. Supporting this interpretation, analysis of fertile spike number in our trials revealed that the triple mutant produced ∼9% fewer spikes across Experiments 1–3 (Fig. 1; Supplementary Table S5). A lack of any genotype×environment interaction for final grain yield suggests that compensatory effects between grain number and weight in these lines occurs predictably, independent of growing conditions. Therefore, *GW2* represents a reliable modifier of grain size but is unlikely to drive yield improvements without additional genetic interventions or agronomic management strategies that restore tiller number.

Given the observed discrepancy between spike- and canopy-level yield, we considered whether focusing solely on main tillers for spike yield component analysis may have biased the results. However, evaluation of both main and secondary spikes in Experiments 5 and 6 showed that they responded similarly; higher TGW, fewer grains per spike, and increased spike yield (Fig. 3). Although the magnitude of these effects was slightly smaller in secondary spikes, yield per spike remained consistently higher, indicating that the reduction in total grain yield observed in *GW2* mutants primarily reflects a lower number of tillers/spikes rather than reduced spike-level productivity. This interpretation is consistent with previous studies (Jian *et al*., 2024; Kis *et al*., 2024; Vicentin and Calderini, 2025, Preprint) where *GW2* was also reported to play a role in regulating tiller number.

The stability of *GW2*-related phenotypes across extreme sowing densities (Experiment 5) suggests that the underlying developmental processes are largely independent of plant competition or tillering dynamics. Despite large differences in tiller number driven by seed rate, the relative increase in TGW and reduction in grain number were consistent, supporting the idea that *GW2* acts directly on grain formation rather than through alteration of assimilate competition among tillers or spikes (Fig. 2; Supplementary Table S7). Although tiller number late in development was consistently lower in the triple mutant, the number of tillers per plant did not differ significantly, and the rate of tiller abortion was also comparable. This indicates that the lower spike number in *GW2* mutants is likely to result from reduced tiller initiation or poorer establishment, rather than increased tiller abortion or reduced tillering capacity *per se*. The observed reduction in plant establishment in the *GW2* triple mutant, particularly under high sowing densities supports this (Supplementary Table S7).

*GW2* is primarily characterized as a negative regulator of cell division influencing grain size, whereas its broader role in developmental processes is less defined. Although it is most highly expressed in the developing grain, it is also expressed at moderate levels in vegetative tissues (shoots and leaves) and could therefore plausibly influence tiller initiation. Quantitative studies have previously demonstrated the negative relationship between grain size and grain number, through either spikes per unit area or grain number per spike (Xie and Sparkes, 2021). Although tiller initiation occurs earlier in development than grain filling, tiller number will influence grain number through its increased investment in reproductive sinks which can limit available assimilates to initiated tillers. Although genotype did not significantly affect tiller abortion at any of the three seed rates, we detected a significant genotype×density interaction, driven primarily from elevated levels at the standard rate.

The well-established trade-off between grain number and grain size in wheat reflects a fundamental, but not total, physiological limitation, demonstrating that this relationship can be uncoupled. For example, ectopic expression of the expansin gene *TaExpA6* in developing wheat grains (Calderini *et al*., 2021) increased grain weight without reducing grain number, thereby overcoming the trade-off. Likewise, recent work has characterized the *TaGW2–TaSPL14* regulatory module, which influences the balance between tiller number and grain weight in wheat (Jian *et al*., 2024). *TaSPL14* negatively regulates tillering but positively affects grain weight, providing opposite effects to *TaGW2*. Overexpression of *TaSPL14* reduced tiller number and increased grain size, whereas its knockout had the opposite effect. *TaGW2* was shown to be an interacting protein of *TaSPL14* with an ability to mediate its ubiquitination and degradation. This provides a mechanistic explanation for reduced tillering in *TaGW2* knockout NILs through decreased ubiquitination and degradation of *TaSPL14*. Collectively, these findings show that although the grain number–grain size trade-off can be explained by resource allocation, it can also be genetically shifted by manipulating key regulatory nodes. Therefore, exploring new allele combinations remains a valid and promising strategy for mitigating the trade-off.

Further study is required to determine the mechanistic effects of *GW2* on establishment and tillering dynamics. A clearer understanding of these subtle physiological effects and their contribution to reduced spike density will be essential for optimizing yield performance in breeding programmes.

Given that the majority of modern wheat cultivars carry semi-dwarfing *RHT-B1b* and *RHT-D1b* alleles, understanding their interaction with *GW2* is useful for breeding. Our data demonstrate that while *RHT* alleles predictably reduced plant height and TGW, *GW2* maintained its grain size phenotype largely independently of *RHT* status (Fig. 4; Supplementary Table S8) . However, some allelic specificity was evident: *RHT-B1b* dampened the positive TGW effects of *GW2* and was associated with yield penalties in the *GW2* mutant background, whereas *RHT-D1b* did not significantly affect productivity. Importantly, discovering that the major positive effects of *GW2* on grain size and spike yield are compatible with *RHT-D1b* suggests that introducing *GW2* mutations into modern semi-dwarf backgrounds should not compromise yield potential.

This study highlights the inherent complexity of yield improvement via a single gene. While *GW2* loss of function consistently enhances grain weight and quality (including higher protein and gluten content), compensatory reductions in grain number and spike number prevent net yield gains. It should be recognized that the lines used here are backcrossed introgression lines derived from tetraploid *T. turgidum* cv Kronos and hexaploid *T. aestivum* cv Cadenza mutant populations into the Paragon background, and potential linkage drag may have influenced observed phenotypes. *GW2_A1* resides within a large haplotype block on chromosome 6A with limited recombination (Brinton *et al*., 2020), which could contribute to linked effects unrelated to *GW2* itself. Furthermore, these results were obtained within a single genetic background (cv. Paragon), and additional studies using alternative genotypes or breeding populations would confirm whether these trade-offs are conserved across different germplasm in distinct environments. Previously, we discovered intervarietal differences in homoeologue effects associated with differences in homoeologue expression levels (Wang *et al*., 2018). However, this comparison should be interpreted in light of differences in genetic background and experimental approach, comparing gene-edited lines in the US cultivar Bobwhite with backcrossed TILLING lines (from Kronos and Cadenza TILLING populations) in the UK cultivar Paragon. Utilizing targeted gene editing approaches could circumvent these issues by generating precise allelic variants directly within elite genetic backgrounds, thereby avoiding linkage drag and confounding background effects. Nevertheless, these mutants represent valuable genetic resources for breeding programmes aiming to manipulate grain morphology.

Equally important is the validation of genetic effects under realistic field conditions. Robust, multi-year and multi-location trials are essential to accurately assess loci influencing yield and yield components. Controlled or single-season experiments often fail to capture the full extent of genotype×environment interactions that determine agronomic performance. Multi-environment trials allow for the separation of genetic and environmental effects (Khaipho-Burch *et al*., 2023) and provide the most reliable measure of trait stability. This is especially critical for yield-associated genes, where compensation among components (grain number, grain weight, and spike number) can often either mask or amplify genetic effects depending on differing conditions. Through the UK Biotechnology and Biological Sciences Research Council (BBSRC) DFW Breeders Toolkit, we accessed multi-location breeder trials that complemented our own experiments. The consistency of *GW2* phenotypes across multiple experiments, including the six breeder trials, provides strong evidence for its robust effects on grain morphology, and emphasizes the need for validation prior to deployment in breeding programmes.

Our results indicate that the *GW2-A1D1* double mutant offers a promising balance between enhanced grain size and limited yield penalty. Across experiments, this genotype produced consistent TGW gains (∼14%) without significant reductions in yield (Fig. 1; Supplementary Table S5), suggesting that partial suppression of *GW2* activity may be sufficient to achieve beneficial increases in grain weight while maintaining grain number. From a breeding perspective, targeting specific homoeologue combinations, particularly *A1D1*, could therefore enable introgression of grain size improvements without compromising productivity. Furthermore, strategically combining *GW2* alleles with loci that increase spikelet fertility or floret survival, such as *TaGNI1* (Sakuma *et al*., 2019) and *TaCKX2.4* (Li *et al*., 2018), or tillering capacity, such as *TaSPL14* (Jian *et al*., 2024) and *TaD27-B* (Zhao *et al*., 2020), may offset reductions in grain number. Future studies should focus on the physiological and molecular basis of this trade-off, including how different *GW2* dosage combinations influence assimilate partitioning and floret fertility. Integrating this knowledge with predictive breeding and genome editing will be key to unlocking the full potential of *GW2* in yield improvement.

## Conclusion

In summary, *TaGW2* acts additively across homoeologues to increase grain size and weight in wheat, and its effects are stable across environments, planting densities, and in the presence of *RHT-D1b*. However, complete loss of *GW2* function results in a consistent trade-off between grain size and number, leading to neutral or slightly negative effects on yield. The *TaGW2-A1D1* double mutant, by contrast, provides a valuable intermediate phenotype, combining increased TGW with maintained yield, and may therefore represent the optimal target for breeding programmes seeking to enhance grain size without incurring yield penalties.

The absence of detrimental interactions between *GW2* and *RHT-D1b* further supports the feasibility of incorporating these alleles into semi-dwarf elite backgrounds. Realizing yield benefits from *GW2* modification will probably require complementary alleles or agronomic interventions that restore spike number or enhance source capacity, thereby capitalizing on the larger sink potential conferred by *GW2* mutations.

## Supplementary Material

erag326_Supplementary_Data

## Data Availability

All raw data have been deposited and are available at Zenodo, 10.5281/zenodo.17778692 (Simmonds *et al*., 2026).

## Supplementary data

The following supplementary data are available at *JXB* online.

Table S1. *GW2* mutation used in this study.

Table S2. KASP markers and primer sequences used in this study.

Table S3. Field trials information.

Table S4. A list of traits phenotyped for each field trial experiment.

Table S5. Summary analysis table of Paragon *GW2* NILs evaluated in Experiments 1–3.

Table S6. Summary analysis table of Paragon *GW2* NILs evaluated in multi-site breeders trials (Experiment 4).

Table S7. Summary analysis table of Paragon *GW2* NILs evaluated at three different seed rates in Experiment 5.

Table S8. Summary analysis table of Paragon *GW2×RHT* NILs evaluated in Experiment 6.
